# Estimation of the burden of varicella in Europe before the introduction of universal childhood immunization

**DOI:** 10.1186/s12879-017-2445-2

**Published:** 2017-05-18

**Authors:** Margarita Riera-Montes, Kaatje Bollaerts, Ulrich Heininger, Niel Hens, Giovanni Gabutti, Angel Gil, Bayad Nozad, Grazina Mirinaviciute, Elmira Flem, Audrey Souverain, Thomas Verstraeten, Susanne Hartwig

**Affiliations:** 1 P95 Pharmacovigilance and Epidemiology Services, Koning Leopold III Laan 1, 3001 Leuven, Belgium; 2 Division of Paediatric Infectious Diseases and Vaccinology, University of Basel Children’s Hospital, CH-4056 Basel, Switzerland; 3 Interuniversity Institute for Biostatistics and statistical Bioinformatics, Hasselt University, Antwerp, Belgium; 4 Centre for Health Economics Research and Modelling Infectious Diseases and Centre for the Evaluation of Vaccination, Vaccine & Infectious Disease Institute, University of Antwerp, Antwerp, Belgium; 5 Department of Medical Sciences, University of Ferrara, Ferrara, Italy; 6 Universidad Rey Juan Carlos, Madrid, Spain; 7 Department of Primary Care and Public Health, Imperial College London, London, UK; 8 Department of Infectious Disease Epidemiology and Modelling, Norwegian Institute of Public Health, Oslo, Norway; 9 Aixial, 4 rue Danjou, 92513 Boulogne-Billancourt, France; 10 Sanofi Pasteur MSD, 162 avenue Jean Jaurès, 69007 Lyon, France

**Keywords:** Varicella, Disease burden, Europe

## Abstract

**Background:**

Varicella is generally considered a mild disease. Disease burden is not well known and country-level estimation is challenging. As varicella disease is not notifiable, notification criteria and rates vary between countries. In general, existing surveillance systems do not capture cases that do not seek medical care, and most are affected by underreporting and underascertainment. We aimed to estimate the overall varicella disease burden in Europe to provide critical information to support decision-making regarding varicella vaccination.

**Methods:**

We conducted a systematic literature review to identify all available epidemiological data on varicella IgG antibody seroprevalence, primary care and hospitalisation incidence, and mortality. We then developed methods to estimate age-specific varicella incidence and annual number of cases by different levels of severity (cases in the community, health care seekers in primary care and hospitals, and deaths) for all countries belonging to the European Medicines Agency (EMA) region and Switzerland.

**Results:**

In the absence of universal varicella immunization, the burden of varicella would be substantial with a total of 5.5 million (95% CI: 4.7–6.4) varicella cases occurring annually across Europe. Variation exists between countries but overall the majority of cases (3 million; 95% CI: 2.7–3.3) would occur in children <5 years. Annually, 3–3.9 million patients would consult a primary care physician, 18,200–23,500 patients would be hospitalised, and 80 varicella-related deaths would occur (95% CI: 19–822).

**Conclusions:**

Varicella disease burden is substantial. Most cases occur in children <5 years old but adults require hospitalisation more often and are at higher risk of death. This information should be considered when planning and evaluating varicella control strategies. A better understanding of the driving factors of country-specific differences in varicella transmission and health care utilization is needed. Improving and standardizing varicella surveillance in Europe, as initiated by the European Centre for Disease Prevention and Control (ECDC), is important to improve data quality to facilitate inter-country comparison.

**Electronic supplementary material:**

The online version of this article (doi:10.1186/s12879-017-2445-2) contains supplementary material, which is available to authorized users.

## Background

Varicella Zoster Virus (VZV) is a double-stranded DNA virus of the herpes virus family [1]. It causes varicella (chickenpox), a highly communicable disease which is usually contracted in early childhood, typically affecting children 2–8 years of age [1]. Varicella is usually a mild disease, but can cause complications requiring hospitalisation [2, 3] and, in rare instances, can even be fatal [4]. After initial infection with VZV, the virus becomes latent in sensory nerve ganglia. Viral reactivation, which usually occurs with increased age or immunosuppression, causes herpes zoster (shingles). Shingles is a painful condition associated with complications including post-herpetic neuralgia and cerebrovascular disease [1].

Although several vaccines for the prevention of varicella are licensed in the European Union (EU), few EU member states (*n* = 7) have implemented a general recommendation for their use [4]. This may be related to a lack of data on the epidemiology of varicella at the country level. The estimation of varicella burden at country level is challenging. Varicella is not a mandatory reportable disease in the EU [4], and systematic pan-European surveillance does not exist. Data, if existing, are based either on national mandatory reporting or more rarely, on national sentinel surveillance systems [5]. The systems differ by the type of cases captured (all cases vs. medically attended cases or only cases with complications), case definitions used, methods for case ascertainment (clinical, laboratory, epidemiologically-linked, or combinations thereof), and data type (case-based or aggregated data). Additionally, available surveillance systems are almost all affected by underreporting [6, 7] and underascertainment: most surveillance systems only capture medically attended disease but not all patients with varicella seek medical care [8].

Systematic literature reviews (SLRs) on the burden of varicella in the EU have recently been conducted by ECDC [4] and Helmuth et al. [3], but like previous reviews, they were descriptive in nature. We set out to quantify the country-specific burden of varicella disease in Europe by using all publicly available data and extrapolating for those countries where we did not find data. To our knowledge, our study is the first to systematically estimate the burden of varicella for individual European countries. We anticipate that this work will contribute to a better understanding of the burden of varicella in Europe, and support decision-making regarding varicella vaccination.

## Methods

### Systematic literature review

#### Search strategy

A PubMed search was conducted for peer-reviewed publications reporting primary incidence, mortality or seroprevalence data in any language in countries under the European Medicines Agency (EMA) plus Swizerland. The search was limited to articles published on or after January 1st, 1995 and restricted to human studies. The full search string used was “Varicella AND (mortality OR complications OR epidemiology OR seroprevalence OR prevalence OR incidence) AND (“Europe”[Mesh] OR Austria OR Belgium OR Bulgaria OR Croatia OR Cyprus OR “Czech Republic” OR Denmark OR Estonia OR Finland OR France OR Germany OR Greece OR Hungary OR Iceland OR Ireland OR Italy OR Latvia OR Liechtenstein OR Lithuania OR Luxembourg OR Malta OR Netherlands OR Norway OR Poland OR Portugal OR Romania OR Slovenia OR Slovakia OR Spain OR Sweden OR “United Kingdom” OR Switzerland)”. Additional information was obtained from the ECDC and national health institutes websites, and through personal communication with national varicella surveillance focal points. Hand searching of the reference lists of papers selected for inclusion was conducted to identify additional publications.

#### Outcomes

Outcomes of interest for the literature review were varicella IgG antibody seroprevalence, incidence of varicella (varicella associated primary care visits and hospitalisations) and mortality.

#### Eligibility criteria

Studies were eligible for inclusion if: 1) they provided data for one or more of the outcomes of interest in the general population, 2) the data were collected before the introduction of universal varicella immunization for countries where universal varicella immunization has been introduced, and 3) the study was published on or after January 1st 1995. Studies were excluded if they did not contain primary data or if the study population was not representative of the general population in terms of varicella transmission dynamics and/or risk of infection (e.g. immunosuppressed patients, imprisoned individuals, or day care workers).

#### Data extraction

Two reviewers (MB, MR) screened titles and abstracts. Discrepancies were extensively discussed and no third reviewer was necessary to resolve disagreements. Evaluation of full text eligibility and data extraction was conducted by a single reviewer (MR). For articles published in languages other than English, reviewers were able to directly read and extract articles in Dutch, German, French, Swedish, Italian, Portuguese and Spanish. For one article in Icelandic, translation software was used for the body text. Legends for the tables and the abstract were provided in English by the journal. For quality control, a sample of 10% of the papers was re-extracted by a third reviewer (TV). The following data was extracted and stored in an MS Excel grid when available: author, journal, year of publication, country, study design, setting (community, primary care, hospital, other), population, case ascertainment, age range, sample size, and incidence or proportion with 95% confidence intervals (CIs). The quality of the evidence was assessed by a single reviewer (MR) with a risk of bias tool adapted from the one by Hoy et al. [9] (Additional file 1). This tool facilitated the scoring of studies from 0 to 8, with the following four categorisations: Excellent (very low risk of bias) – score 8 and prospective study design; Good (low risk of bias) – score 8, but no prospective study design; Acceptable (medium risk of bias) score 6–7; Low (high risk of bias) – score less than 6.

### Incidence estimation

We aimed to estimate the annual incidence rates of varicella cases that 1) occurred in the community (with or without health care visit), 2) resulted in an ambulatory primary health care visit, 3) required hospitalisation, or 4) caused death.

Data sources were used for incidence estimation if: 1) studies attained a quality score of 6 or more; 2) data collection was conducted for at least 1 year; and 3) age-specific data was provided. In cases where the same data source was used to report incidence for different time periods, we selected the source with the longest time period covered and/or most recent data for inclusion in our analyses. We excluded data derived from mandatory notification systems which may be substantially affected by underreporting [10], except for countries where mandatory notification was the only data source available. When the age groups reported in the original data source did not match our age groups of interest, we either used weighted averages (to combine several age groups) or we redistributed the age groups assuming constant incidence within the age groups (to split a single age group). Age groups without upper limits were excluded from the calculations, because the width of the age group was not known and therefore the incidence cannot be recalculated for the age group of interest.

### Incidence estimation for countries with data

Few studies in Europe have assessed the incidence rate of varicella at the community level. We therefore opted to derive the annual age-specific varicella incidence (per 100,000) from seroprevalence studies, which are commonly available. Particularly, for each country we first estimated age-specific seroprevalence using the catalytic model with a piecewise constant force of infection. Then, from these estimated profiles, we derivedincidence rates (and 95% CIs) as differences in seroprevalences for six age groups, < 5, 5–9, 10–14, 15–19, 20–39 and 40+ years. This approach assumes lifelong immunity, time homogeneity and non-differential mortality. For more details on the methodology used, we refer to Bollaerts et al. (Bollaerts K, Riera-Montes M, Hens N et al. A systematic review of varicella seroprevalence in European countries before universal childhood immunization: deriving incidence from seroprevalence data. Submitted 2017).

For incidence rates at the primary care and hospital level, we relied upon the published age-specific data for countries with more extensive information. For countries with more than one estimate, we provide a range with the lowest and highest estimates.

Varicella-specific mortality data was obtained from the World Health Organisation (WHO) European Detailed Mortality Database (DMDB) [11]. The DMDB contains mortality data by cause of death (ICD-9 or ICD-10 codes), age and sex. Other country-specific mortality data sources identified during the SLR were found either to rely upon the same data source that feeds into the WHO DMDB or to provide comparable results. We obtained mortality data for all countries for the 10 most recent years available prior to the introduction of universal varicella immunization. We calculated the Poisson exact 95% CIs of the mortality rates.

### Incidence estimation for countries without data

To obtain age-specific community, primary care and hospital incidence estimates for countries without data, we took a 2-step approach. Firstly, we tried to build prediction models for each outcome and age group, calibrated using information from the countries with data. Secondly, when the model failed to predict (i.e. non-significance of any of the potential predictor variables), we extrapolated the minimum and maximum observed estimates within the same age group from countries with data (Table 2).

Particularly, we built a linear regression model based on the incidences in the younger age groups and country-level prediction variables that have been shown to be associated with country-level differences in varicella transmissibility and health care use [12, 13] (Additional file 2): proportion of children <3 years that receive no formal childcare [14], population density [14], inequality in income distribution [14], proportion of people at risk of poverty [14], total health expenditure [15], proportion of households with 1, 2, 3, and 4 or more children [14], number of annual consultations of a medical doctor per inhabitant [14], and number of acute hospital discharges per 100 population [14].

We failed to predict primary care incidence (PCI) and hospitalisation incidence (HI). Therefore, we relied upon the ratio between PCI and community incidence - the primary care rate (PCR), and on the ratio between HI and community incidence - the hospitalisation rate (HR). Specifically, we calculated PCI or HI by multiplying the country’s age-specific community incidence with the corresponding age-specific average minimum-maximum observed PCR or HR. We preferred this approach to the alternative approach of simply imputing the PCI and HI that was observed in other countries, as this would not take into account differences in community incidence.

No imputations were required for mortality data, as these were available from the DMDB for all countries.

### Validation

To evaluate our methodology, we used information from a recent varicella study in Norway [16], which was conducted in 2015 and published after our literature search had concluded. This study provided both age-specific seroprevalence proportions and age-specific estimates of PCI at the national level. We used this published seroprevalence data to estimate varicella community incidence and derive PCI as described above.

### Estimation of annual number of cases

To estimate the annual country-specific numbers of varicella-associated community cases, ambulatory primary care consultations, hospitalisations, and deaths per age group, we applied age-specific community incidence, PCI, HI, and mortality rates to each country’s population. Population data was obtained from Eurostat for the latest year available (2015) [14]. The total annual number of cases within Europe was then calculated by summing the number of cases for each country. The numbers of varicella-associated community cases and deaths are presented as point estimates with 95% CIs, while the numbers of varicella-related primary care consultations and hospitalisations are presented as a range.

We compared the estimated annual country-specific numbers of varicella-associated community cases to the numbers reported to EUVAC.NET, in order to estimate the underreporting of the disease. EUVAC.NET is a European surveillance network for selected vaccine-preventable diseases that was active until 2011 and was hosted by the Staten Serum Institute (SSI), Denmark. It incorporated 18 countries; all EU Member States up to 2011, as well as Croatia, Iceland, Norway, Switzerland and Turkey. Number of varicella cases were reported annually by all countries. We compared the EUVAC.NET data of 2009–2010 to our results, and calculated the ratio of reported to estimated number of cases, expressed in percentages.

## Results

### Systematic literature review

The literature search was conducted in PubMed on October 2nd 2015. Identification of grey literature sources was conducted between October and December 2015.

**Fig. 1:**
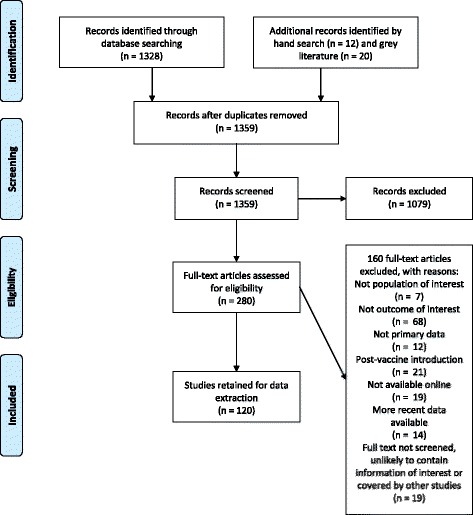
PRISMA Flow Diagram

We identified 120 data sources from 31 countries for extraction (Fig. 1) [5, 7, 8, 11, 17–127]. Most data sources (97/120) scored the maximum in terms of quality assessment (score of 8). Only one data source scored less than 6. A table summarizing the main characteristics of all selected data sources, and a summarised version of the full data extraction table is provided in Additional files 3 and 4.

The most frequently found unique data source was on seroprevalence (*n* = 52) [17–68], followed by hospitalisation (*n* = 39) [23, 27, 40, 69–104], primary care (*n* = 27) [7, 8, 23, 27, 74, 76, 78–81, 97, 99, 102, 104–117], mortality (*n* = 15) [11, 23, 27, 74–76, 79, 80, 84, 91, 97, 118–121], and incidence of reported varicella cases through mandatory surveillance (*n* = 14) (Table 1) [5, 26, 27, 40, 79, 80, 101, 116, 122–127]. Varicella community incidence was estimated from 43 seroprevalence data sources from 16 countries [17–27, 29–41, 44–47, 49–54, 57–62, 64, 65, 68], primary care incidence from 17 PCI data sources from 8 countries [23, 74, 76, 79, 80, 99, 101, 102, 104–106, 109, 110, 112, 114, 124, 126], hospitalisation incidence from 18 HI data sources from 10 countries [23, 69–73, 75, 76, 79–81, 84, 87, 90, 91, 94, 102, 103], and one data source was used to estimate mortality in 31 countries [11] (Table 2). Data on all four outcomes was only available for six countries (Belgium, France, Italy, the Netherlands, Spain and the UK).

**Table 1:** Number of literature sources available per outcome per country, broken down by outcome^a^

Country	N of sources		Outcome			
Country	N papers	N grey literature	Sero-prevalence	Primary care incidence	Hospitalization incidence	Mortality
Austria	1	1	1			1
Belgium	5	4	4	3	2	3
Bulgaria	0	4		3		1
Croatia	1	4	1	3		1
Cyprus	0	3		2		1
Czech Rep	0	4		3		1
Denmark	0	1				1
Estonia	0	4		3		1
Finland	4	4	3	3		2
France	6	7	3	6	3	4
Germany	7	1	2	2	2	2
Greece	4	2	1	1	3	1
Hungary	1	4		4		1
Iceland	1	1	1			1
Ireland	4	4	3		3	2
Italy	14	3	7	9	5	2
Latvia	0	3		2		1
Lithuania	0	4		3		1
Luxembourg	1	1	1	1	1	1
Malta	0	4		3		1
Netherlands	7	2	4	5	4	2
Norway	0	1				1
Poland	3	4	1	5	1	1
Portugal	3	1	2	1		1
Romania	1	4		4		1
Slovakia	1	4	1	3		1
Slovenia	2	4	1	4		1
Spain	22	2	10	5	9	3
Sweden	4	1	1		3	1
Switzerland	6	1	5	1		1
UK	13	4	5	12	3	4
*Total*	101	18	57	91	39	46
*Unique data sources*			52	37	39	15

^a^This includes literature sources that were not eligible for the estimation of the burden of varicella disease in Europe

**Table 2:** Number of data sources and countries included in incidence estimation per outcome

Outcome	N data sources	N countries	Countries with data
Community incidence (derived from seroprevalence data)	43	16	Belgium, Finland, France, Germany, Greece, Iceland, Ireland, Italy, Luxembourg, Netherlands, Poland, Slovakia, Slovenia, Spain, Switzerland, UK
Primary care incidence	17	8	Belgium, France, Italy, Netherlands, Poland, Romania, Spain, UK
Hospitalization incidence	18	10	Belgium, France, Germany, Greece, Ireland, Italy, Netherlands, Spain, Sweden, UK
Mortality	1	31	All

### Varicella community incidence

The final model to predict community incidence in <5 year olds included as country-level predictors the proportion of children <3 years attending pre-school care and population density, with a moderate goodness of fit (R^2^ = 40%). For 5–9 year olds, the prediction model included the incidence in <5 year olds with a moderate to high goodness of fit (R^2^ = 80%). For the older age groups, we had to rely upon extrapolation. We first categorised countries based on the speed of varicella acquisition in children <5 years (annual incidence < or ≥10%) as in Bollaerts et al. (Bollaerts K, Riera-Montes M, Hens N et al. A systematic review of varicella seroprevalence in European countries before universal childhood immunization: deriving incidence from seroprevalence data. Submitted 2017). Then, we extrapolated the minimum and maximum age specific community incidence observed in countries with data (Table 2) to countries without data within the same category.

Age-specific annual incidence rates of varicella, as derived from serological data, varied considerably across the countries, particularly in age groups 10–14 and 15–19 years. In most countries the highest annual incidence was observed amongst children aged <5 years, ranging from 7052 (Greece) to 17,974 (Malta) per 100,000 (Table 3). In Bulgaria, Czech Republic, Italy, Romania, Switzerland and Greece the highest incidence was observed in 5–9 year olds. From the age of 10 years onwards, varicella incidence dropped drastically. Countries with the highest incidence in children <5 years had lower incidence rates in 10–14 year olds than those countries with the highest incidence in children 5–9 years of age. By the age of 15–19 years, incidence was <1000 per 100,000 in all countries with the exception of Greece.

**Table 3:** Age-specific annual community incidence (/100,000) of varicella in European countries before the introduction of universal childhood immunization programs

Country	Annual incidence /100.000 (95% CI) per age group in years					
	<5	5–9	10–14	15–19	20–39	40+
Austria^a^	8986 (7449–10,523)	8421 (7643–9200)	1267 (882–1652)	688 (554–822)	195 (192–198)	18 (10–26)
Belgium	14,628 (13,848–15,408)	4126 (3160–5124)	284 (136–480)	220 (114–342)	120 (75–163)	38 (31–45)
Bulgaria^a^	8097 (6237–9957)	9053 (8136–9970)	1267 (882–1652)	688 (554–822)	195 (192–198)	18 (10–26)
Croatia^a^	8911 (7386–10,436)	8475 (7685–9264)	1267 (882–1652)	688 (554–822)	195 (192–198)	18 (10–26)
Cyprus^a^	10,039 (8838–11,240)	7674 (7022–8325)	272 (0–916)	213 (0–698)	123 (0–372)	48 (0–113)
Czech Republic^a^	7707 (5350–10,065)	9330 (8346–10,313)	1267 (882–1652)	688 (554–822)	195 (192–198)	18 (10–26)
Denmark^a^	15,965 (12,545–19,385)	3464 (2307–4622)	272 (0–916)	213 (0–698)	123 (0–372)	48 (0–113)
Estonia^a^	8850 (7256–10,444)	8518 (7720–9317)	1267 (882–1652)	688 (554–822)	195 (192–198)	18 (10–26)
Finland	10,130 (9336–10,850)	8680 (7830–9530)	40 (0–102)	38 (0–94)	35 (0–79)	30 (0–55)
France	13,488 (12,698–14,254)	4668 (3554–5722)	554 (260–974)	388 (208–596)	172 (113–222)	36 (17–56)
Germany	11,884 (10,972–12,646)	7048 (6192–8046)	246 (156–374)	190 (126–266)	103 (77–128)	32 (24–40)
Greece	7052 (5986–7998)	7462 (5026–9578)	2804 (0–6364)	1370 (0–1666)	310 (0–543)	14 (0–213)
Hungary^a^	8752 (7101–10,404)	8588 (7775–9401)	1267 (882–1652)	688 (554–822)	195 (192–198)	18 (10–26)
Iceland	11,460 (8400–12,520)	7940 (6840–11,260)	0 (0–0)	0 (0–0)	0 (0–0)	0 (0–0)
Ireland	11,954 (10,688–13,194)	6434 (4894–7776)	76 (0–412)	72 (0–334)	65 (0–209)	52 (0–91)
Italy	8020 (7320–8736)	8118 (7072–9112)	916 (666–1242)	698 (534–892)	372 (320–421)	113 (93–128)
Latvia^a^	9239 (7726–10,751)	8242 (7498–8986)	1267 (882–1652)	688 (554–822)	195 (192–198)	18 (10–26)
Lithuania^a^	9468 (8045–10,891)	8079 (7365–8794)	1267 (882–1652)	688 (554–822)	195 (192–198)	18 (10–26)
Luxembourg	15,720 (14,482–16,790)	3292 (2070–4560)	152 (58–314)	128 (54–238)	86 (44–126)	41 (28–49)
Malta^a^	17,974 (7481–28,467)	2037 (489–3586)	272 (0–916)	213 (0–698)	123 (0–372)	48 (0–113)
Netherlands	16,122 (15610–16,592)	3350 (2854–3902)	20 (4–46)	20 (4–42)	18 (4–36)	15 (3–26)
Norway^a^	13,279 (10,433–16,124)	5372 (4653–6092)	272 (0–916)	213 (0–698)	123 (0–372)	48 (0–113)
Poland	8974 (7980–9990)	7734 (6148–9192)	1652 (662–2740)	822 (492–1022)	192 (94–284)	10 (1–71)
Portugal^a^	12,693 (11,009–14,378)	5788 (5134–6443)	272 (0–916)	213 (0–698)	123 (0–372)	48 (0–113)
Romania^a^	7108 (4663–9552)	9755 (8665–10,846)	1267 (882–1652)	688 (554–822)	195 (192–198)	18 (10–26)
Slovakia	9362 (8398–10,330)	8264 (6926–9564)	882 (462–1494)	554 (348–758)	198 (152–230)	26 (8–56)
Slovenia	11,640 (10,764–12,472)	6954 (5978–7956)	274 (148–464)	220 (128–336)	132 (93–169)	51 (36–61)
Spain	10,874 (10,234–11,550)	7312 (6572–8014)	314 (236–400)	260 (202–318)	165 (139–188)	71 (64–76)
Sweden^a^	13,578 (10,635–16,522)	5160 (4401–5919)	272 (0–916)	213 (0–698)	123 (0–372)	48 (0–113)
Switzerland	7368 (6494–8314)	11,798 (10,816–12,722)	74 (0–346)	68 (0–220)	54 (0–94)	36 (0–42)
UK	12,982 (12,230–13,718)	4656 (3638–5610)	388 (160–696)	324 (148–530)	212 (120–281)	95 (74–106)

^a^countries where community incidence was predicted

### Varicella primary care incidence

We failed to model the PCI for any of the age groups and we therefore had to rely upon the maximum-minimum approach. The estimated PCRs ranged from 18% (Netherlands, 5–9 year olds) to 100% (Netherlands, 10–64 y; Italy, 10–14 y; Spain, 10–14 y; UK, 10–39 y; France, 5–64 y) across all age groups.

Varicella primary care incidence was highest in children <5 years with substantial inter-country variation, ranging from 1100 per 100,000 in Romania to 13,069 per 100,000 in France. Incidence decreased with age, dropping substantially from the age of 10 years onwards (Table 4).

**Table 4:** Age-specific annual primary care incidence/100,000 of varicella in Europe before the introduction of universal childhood immunization programs

Country	Annual incidence /100,000 (min-max) per age group in years					
	<5	5–9	10–14	15–19	20–39	40+
Austria^a^	4773–5322	3623–5076	1610–3457	454–982	136–251	11–18
Belgium	4502-NA	1006-NA	190-NA	63-NA	49-NA	15-NA
Bulgaria^a^	4301–4796	3895–5457	1610–3457	454–982	136–251	11–18
Croatia^a^	4733–5278	3646–5109	1610–3457	454–982	136–251	11–18
Cyprus^a^	5332–5946	3302–4626	346–742	141–304	86–158	30–49
Czech Rep.^a^	4093–4565	4014–5624	1610-3457	454–982	136–251	11–18
Denmark^a^	8480–9456	1490–2088	346–742	141–304	86–158	30–49
Estonia^a^	4701–5242	3665–5134	1610–3457	454–982	136–251	11–18
Finland^a^	5380-6000	3735-5232	51-109	25–54	24–45	19–31
France	10,694–13,069	3344–5917	368–1283	161–342	68–281	28–56
Germany^a^	6312-7039	3032-4248	313-671	125–271	72–133	20–33
Greece^a^	3746-4177	3211-4498	3562-7651	904–1955	216–398	9–15
Hungary^a^	4649–5184	3695–5177	1610–3457	454–982	136–251	11–18
Iceland^a^	6087-6788	3416-4786	0-0	0–0	0–0	0–0
Ireland^a^	6349-7080	2768-3878	97-207	48–103	45–83	33–53
Italy	7476–7582	4548–5062	1052–2283	461–996	260–478	71–116
Latvia^a^	4907–5472	3546–4968	1610–3457	454–982	136–251	11–18
Lithuania^a^	5029–5608	3476–4870	1610–3457	454–982	136–251	11–18
Luxembourg^a^	8350-9311	1416-1984	193-415	84–183	60–111	26–42
Malta^a^	9547–10,646	876–1228	346–742	141–304	86–158	30–49
Netherlands	3032–5817	591–1535	52–180	32–92	23–50	12–23
Norway^a^	7053–7865	2311–3238	346–742	141–304	86–158	30–49
Poland	3929-NA	4106-NA	923-NA	197-NA	77-NA	9-NA
Portugal^a^	6742–7518	2490–3489	346–742	141–304	86–158	30–49
Romania	1100-NA	1450-NA	1000-NA	500-NA	89-NA	8-NA
Slovakia^a^	4973-5545	3556-4981	1121-2407	366–791	138–255	17–27
Slovenia^a^	6182-6894	2992-4192	348-748	145–314	92–170	32–52
Spain	8304-NA	3281-NA	747-NA	172–371	115–212	45–73
Sweden^a^	7212–8042	2220–3110	346–742	141–304	86–158	30–49
Switzerland^a^	3913-4364	5076-7112	94-202	45–97	38–69	22–37
UK	3838–4695	1562–3012	339–653	245–365	213–255	22–67

^a^countries where primary care incidence was predicted NA: Not applicable

### Varicella hospitalisation incidence

We also failed to model the HI for any age group and used the minimum-maximum approach instead. The estimated HRs ranged from 0.05% (Ireland, 5–9 y) to 3.5% (Netherlands, 20–39 y) across all age groups.

All countries presented the highest HI in children <5 years. Incidence ranged from 9 per 100,000 in Sweden to 75 per 100,000 in France, decreasing to <20 per 100,000 for all countries in the 5–9 years age group (Table 5). In older age groups, HI remained ≤10 per 100,000.

**Table 5:** Age-specific annual hospitalization incidence (/100,000) of varicella in Europe before the introduction of universal childhood immunization programs

Country	Annual incidence /100,000 (min-max) per age group in years					
	<5	5–9	10–14	15–19	20–39	40+
Austria^a^	28–34	10–13	6–9	3–5	3–3	0–0
Belgium	79-NA	3-NA	1–2	1–2	2–2	0–1
Bulgaria^a^	25–31	11–14	6–9	3–5	3–3	0–0
Croatia^a^	28–34	10–13	6–9	3–5	3–3	0–0
Cyprus^a^	31–38	9–12	1–2	1–2	2–2	1–1
Czech Republic^a^	24–29	11–15	6–9	3–5	3–3	0–0
Denmark^a^	50–60	4–5	1–2	1–2	2–2	1–1
Estonia^a^	28–33	10–13	6–9	3–5	3–3	0–0
Finland^a^	32-38	11–14	0–0	0–0	0–1	0–0
France	57–75	3–9	0.6–1.8	5.6-NA	3.5–NA	1–NA
Germany	45–NA	10–NA	2–NA	1–1	1–2	0–0
Greece	22–NA	12–NA	2.3–NA	7–10	4–5	0–0
Hungary^a^	27–33	10–13	6–9	3–5	3–3	0–0
Iceland^a^	36–43	10–12	0–0	0–0	0–0	0–0
Ireland	10–NA	3-NA	0.9–NA	0.1-NA	0.5–NA	0.4–NA
Italy	37–NA	19–NA	5.2–NA	2.5-NA	2.7–NA	0.7–NA
Latvia^a^	29–35	10–13	6–9	3–5	3–3	0–0
Lithuania^a^	29–36	10–13	6–9	3–5	3–3	0–0
Luxembourg^a^	49-59	4–5	1–1	1–1	1–1	0–1
Malta^a^	56–68	2–3	1–2	1–2	2–2	1–1
Netherlands	15–19	2–2	0.2–0.3	0.1–0.2	0.6–0.6	0.2–0.3
Norway^a^	41–50	7–8	1–2	1–2	2–2	1–1
Poland^a^	28–34	9–12	8–11	4–6	3–3	0–0
Portugal^a^	39–48	7–9	1–2	1–2	2–2	1–1
Romania^a^	22–27	12–15	6–9	3–5	3–3	0–0
Slovakia^a^	29–35	10–13	4–6	3–4	3–3	0–0
Slovenia^a^	36–44	8–11	1–2	1–2	2–2	1–1
Spain	23–51	5–12	0.9–3.1	0.9–NA	2.1–3.1	1–1.6
Sweden	9–30	6–NA	1.2–NA	1–2	2–2	1–1
Switzerland^a^	23–28	14–18	0–1	0–0	1–1	0–0
UK	39–54	8–16	1.5–3.1	2.7–5.5	2.7–5.7	0.6–1

^a^countries where primary care incidence was predicted

### Varicella mortality

Estimated varicella mortality was very low with an annual incidence <0.2 deaths per 100,000 in all age groups and countries (Table 6). For most countries, the mortality was highest in children <5 years. However, in some countries (Czech Republic, Greece, Ireland, Slovakia), mortality was higher in the 5–9 year age group, and in Lithuania, the mortality peak was found in the 10–14 years age group.

**Table 6:** Age-specific annual mortality incidence (/100,000) of varicella in European countries before the introduction of universal childhood immunization programs

Country	Annual incidence /100,000 (95% CI) per age group in years					
	<5	5–9	10–14	15–19	20–39	40+
Austria	0.08 (0–1.06)	0 (0–0.91)	0 (0–0.89)	0 (0–0.81)	0 (0–0.17)	0 (0–0.09)
Belgium	0.13 (0–0.81)	0.02 (0–0.59)	0.02 (0–0.63)	0 (0–0.58)	0 (0–0.13)	0.02 (0–0.11)
Bulgaria	0.03 (0–1.16)	0 (0–1.08)	0 (0–1.16)	0 (0–1.17)	0 (0–0.2)	0 (0–0.09)
Croatia	0.09 (0–1.97)	0.04 (0–1.82)	0 (0–1.79)	0 (0–1.52)	0.01 (0–0.35)	0 (0–0.16)
Cyprus	0 (0–7.45)	0 (0–7.77)	0 (0–8.15)	0 (0–7)	0 (0–1.32)	0 (0–0.93)
Czech Rep.	0 (0–0.66)	0.02 (0–0.68)	0 (0–0.78)	0 (0–0.8)	0 (0–0.12)	0 (0–0.07)
Denmark	0.06 (0–1.36)	0 (0–1.11)	0 (0–1.11)	0 (0–1.05)	0 (0–0.26)	0 (0–0.13)
Estonia	0 (0–5.11)	0 (0–4.9)	0 (0–5.84)	0 (0–6.17)	0 (0–1.03)	0 (0–0.54)
Finland	0 (0–1.22)	0 (0–1.21)	0 (0–1.26)	0 (0–1.2)	0 (0–0.27)	0.03 (0–0.19)
France	0.07 (0.01–0.22)	0.02 (0–0.13)	0.01 (0–0.11)	0 (0–0.1)	0.01 (0–0.04)	0.02 (0.01–0.05)
Germany	0.03 (0–0.17)	0.01 (0–0.13)	0.01 (0–0.12)	0 (0–0.09)	0 (0–0.03)	0 (0–0.02)
Greece	0 (0–0.73)	0.02 (0–0.71)	0.02 (0–0.73)	0 (0–0.68)	0.03 (0–0.18)	0 (0–0.06)
Hungary	0.06 (0–0.95)	0.02 (0–0.79)	0 (0–0.77)	0 (0–0.71)	0.01 (0–0.15)	0 (0–0.08)
Iceland	0 (0–16.04)	0 (0–16)	0 (0–17.3)	0 (0–16.71)	0 (0–3.97)	0 (0–2.52)
Ireland	0 (0–1.04)	0.05 (0–1.17)	0 (0–1.19)	0 (0–1.26)	0 (0–0.29)	0.06 (0–0.3)
Italy	0.04 (0–0.2)	0.01 (0–0.14)	0.01 (0–0.15)	0 (0–0.14)	0.01 (0–0.04)	0.01 (0–0.02)
Latvia	0 (0–3.78)	0 (0–3.48)	0 (0–3.97)	0 (0–4.15)	0 (0–0.68)	0 (0–0.35)
Lithuania	0 (0–2.47)	0 (0–2.67)	0.06 (0–2.82)	0 (0–2.15)	0 (0–0.49)	0 (0–0.24)
Luxembourg	0 (0–11.45)	0 (0–11.66)	0 (0–11.59)	0 (0–10.99)	0 (0–2.3)	0 (0–1.35)
Malta	0 (0–17.66)	0 (0–18.37)	0 (0–18.16)	0 (0–15.28)	0 (0–3.02)	0 (0–1.69)
Netherlands	0.04 (0–0.5)	0.01 (0–0.42)	0.01 (0–0.39)	0 (0–0.37)	0 (0–0.09)	0.02 (0–0.08)
Norway	0 (0–1.18)	0 (0–1.16)	0 (0–1.2)	0 (0–1.13)	0.01 (0–0.28)	0.07 (0.01–0.27)
Poland	0.03 (0–0.25)	0.03 (0–0.23)	0 (0–0.2)	0 (0–0.19)	0 (0–0.04)	0 (0–0.03)
Portugal	0.03 (0–0.88)	0.03 (0–0.8)	0 (0–0.69)	0 (0–0.67)	0.02 (0–0.18)	0.01 (0–0.08)
Romania	0.09 (0–0.56)	0 (0–0.34)	0.01 (0–0.36)	0 (0–0.34)	0 (0–0.07)	0 (0–0.04)
Slovakia	0 (0–1.31)	0.04 (0–1.39)	0 (0–1.4)	0 (0–1.26)	0 (0–0.22)	0 (0–0.14)
Slovenia	0 (0–3.37)	0 (0–3.56)	0 (0–4.02)	0 (0–3.88)	0 (0–0.67)	0.02 (0–0.36)
Spain	0.06 (0–0.27)	0.02 (0–0.18)	0.01 (0–0.19)	0 (0–0.18)	0.03 (0.01–0.08)	0.01 (0–0.04)
Sweden	0.12 (0–0.86)	0.02 (0–0.69)	0.04 (0–0.79)	0 (0–0.7)	0 (0–0.15)	0.04 (0–0.14)
Switzerland	0.03 (0–0.94)	0.03 (0–0.96)	0 (0–0.92)	0 (0–0.84)	0 (0–0.17)	0.03 (0–0.14)
UK	0.11 (0.03–0.27)	0.03 (0–0.16)	0.01 (0–0.12)	0.01 (0–0.12)	0.02 (0–0.05)	0.05 (0.03–0.08)

### Validation

The observed annual community incidence in children under 10 years of age in Norway was in line with our predictions (8669 vs 9326 per 100,000). However, the model overestimated the number of cases in <5 year olds and underestimated the incidence in 5–9 year olds compared to the observed data (Table 7). The model also predicted substantially higher estimates of varicella PCI in Norway in most age groups compared to observed data, particularly in children under 10 years of age (Table 8).

**Table 7:** Norway: varicella age-specific annual community incidence (/100,000) before the introduction of universal childhood immunization programs, predicted vs observed

Age group (years)	Predicted incidence per 100,000 (95% CI)	Observed incidence per 100,000 (95% CI)
< 5	13,279 (10,433–16,124)	9164 (8100–10,344)
5–9	5372 (4653–6092)	8174 (6526–9644)
10–14	272 (0–916)	240 (70–486)
15–19	213 (0–698)	218 (68–414)
20–39	123 (0–372)	174 (62–287)
≥ 40	48 (0–113)	114 (53–149)

**Table 8:** Norway: Varicella age-specific annual primary care incidence (/100,000) before the introduction of universal childhood immunization programs, predicted versus observed

Age group (years)	Observed incidence per 100,000	Predicted incidence per 100,000	
		Extrapolation from observed community incidence	Extrapolation from predicted community incidence
< 5	2030	4851–5410	7053–7865
5–9	919	3517–4927	2311–3238
10–14	189	305–655	346–742
15–19	104	144–311	141–304
20–39	84	121–223	86–158
≥ 40	14	72–117	30–49

### Annual number of varicella cases

**Fig. 2:**
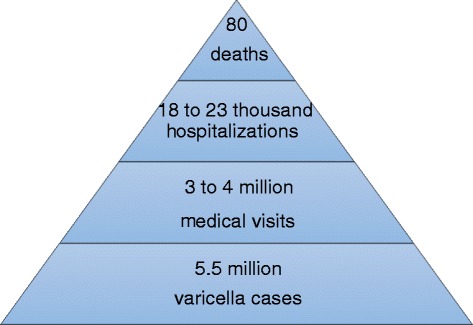
Annual burden of varicella in Europe

We estimated that across European countries, and in the absence of universal varicella immunization, 5.5 million (95% CI: 4.7–6.4) new varicella cases would occur annually. Most cases (3 million; 95% CI: 2.7–3.3) would occur in children <5 years. At least 54% of varicella cases are expected to result in an ambulatory primary care visit and 0.3% will require hospitalisation (Table 9), implying that annually 3–3.9 million patients would consult a primary care physician and 18,200–23,500 patients be hospitalised. In addition, approximately 80 varicella-related deaths are expected to occur every year (95% CI: 19–822) (Fig. 2, Table 9). (See Additional file 5 for country-specific data).

**Table 9:** Annual number of varicella cases, consultations, hospitalizations and deaths and consultation, hospitalization and case fatality rates in Europe before the introduction of universal childhood immunization programs

Outcome	Age group (years)						
	<5	5–9	10–14	15–19	20–39	40+	Total
N varicella cases	3,029,226	1,816,442	175,020	116,926	244,923	141,087	5,523,624
N varicella consultations	1,660,087	826,610	179,400	67,609	164,118	78,015	2,975,839
N varicella hospitalizations	9905	2386	712	718	3209	1233	18,163
N varicella deaths	16	5	2	1	12	45	81
Proportion of varicella cases consulting a physician (%)	54.80	45.51	100.00	57.82	67.01	55.30	53.87
Proportion of varicella cases that are hospitalized (%)	0.33	0.13	0.41	0.61	1.31	0.87	0.33
Case fatality rate (%)	0.001	0.000	0.001	0.001	0.005	0.032	0.001

From comparing the number of annual varicella cases reported to EUVAC.NET to our estimates, an important under-reporting to EUVAC.NET was found, with only <1% (Greece: 6 vs 109,214 cases) to 51% (Slovenia: 11,074 vs 21,729 cases) of all cases of varicella occurring in the community reported to EUVAC.NET (Table 10) [123].

**Table 10:** Number of estimated varicella cases occurring in European countries every year, number of varicella cases reported to EUVAC

Country	Annual estimated number of cases in the community (All ages)	Number of varicella cases reported to EUVAC.NET (2009)	Number of varicella cases reported to EUVAC.NET (2010)	Average number of cases reported to EUVAC.NET (2009/10)	Reported cases/estimated total cases (%)
Bulgaria	68,840	29,117	19,724	24,421	35
Croatia	43,380	17,563	16,027	16,795	39
Cyprus	9387	159	75	117	1
Czech Republic	112,559	47,192	48,270	47,731	42
Estonia	14,846	8556	6146	7351	50
Finland^a^	58,715	360	358	359	1
Greece^b^	109,214	7	5	6	0
Hungary	97,591	40,460	39,602	40,031	41
Italy	597,881	56,502	39,649	48,076	8
Latvia	20,772	5019	3697	4358	21
Lithuania	28,498	12,698	11,042	11,870	42
Malta	4524	183	92	138	3
Norway^c^	63,105	31	NR	31	0
Poland	403,362	140,115	183,446	161,781	40
Romania	206,872	44,693	36,245	40,469	20
Slovakia	57,449	17,735	19,887	18,811	33
Slovenia	21,729	13,060	9087	11,074	51
Spain	478,816	141,399	157,222	149,311	31

*NR* not reported

^a^Finland has a laboratory based surveillance system which does not separate clinical disease and therefore includes both varicella and herpes zoster. Only laboratory confimed cases are reported.^b^Greece: the national mandatory surveillance system includes only varicella cases with complications.^c^Norway: only laboratory confirmed cases of varicella encephalitis reported.

NET during 2009 and 2010, and percentage of reported cases out of the total number of estimated varicella cases occurring in the community

## Discussion

We estimate that in the absence of universal varicella immunization, a total of 5.5 million (95% CI: 4.7–6.4) varicella cases would occur annually across Europe. It has previously been estimated that the annual number of new varicella cases in a country correspond approximately to the size of its birth cohort [4, 27, 94, 128]. Given that according to Eurostat [14] there were 5.2 million live births in Europe in 2015, this is in line with our estimates. Our study estimates that more than half of all varicella cases occur in children <5 years of age, as has been reported previously [4].

We found that community incidence varied greatly between countries, particularly in children and adolescents. This probably reflects different country-specific dynamics in varicella transmission during childhood, which have been associated with differences in social mixing patterns [12, 13]. Countries with low incidence rates in children <5 years of age have higher incidence rates in older age groups. This pattern tends to occur in countries in Eastern and Southern Europe, as has also been observed in a previous review [3].

According to our estimates, most varicella cases (54%) lead to a physician consultation and a small proportion of cases (0.3%) are hospitalised. We found that the highest consultation rates (100%) occurred among children aged 10 to 14 years, while the highest hospitalisation rates (1.3%) were in 20 to 39 year olds. Case fatality rate was highest (0.03%) in the >40 years age group followed by the 20 to 39 years age group (0.005%). These findings confirm that the majority of disease burden is in the younger age groups, but disease is more severe in adults and the elderly [4].

The main strength of our study is that we followed a systematic approach to quantify age-specific varicella incidence. In this way, we maximised transparency and comparability across countries. We based our estimates on the best available evidence, as obtained through a comprehensive SLR of the epidemiology of varicella. To estimate varicella incidence at community level we used seroprevalence data. Unlike other surveillance data, seroprevalence data are not affected by health care seeking or under-reporting and therefore provide a more accurate representation of the incidence at the community level, although using seroprevalence data requires the assumption of time homogeneity (Bollaerts K, Riera-Montes M, Hens N et al. A systematic review of varicella seroprevalence in European countries before universal childhood immunization: deriving incidence from seroprevalence data. Submitted 2017). Seroprevalence data are robust and have previously been used to estimate varicella incidence in Luxembourg, Italy and Spain [10, 40, 129].

Our study has several limitations. Data sources providing PCI, HI and mortality may be affected by under-ascertainment and underreporting, and we may therefore have underestimated the number of primary care visits, hospitalisations and deaths. Some of the studies we used in our estimations were regional only and might not be representative for the respective whole country. Despite our attempts to be comprehensive we cannot guarantee that all relevant data sources were identified in this review. However, we expect the number of missed data sources to be low. We did not find data for all outcomes for all countries, so we extrapolated the incidence for these countries based on data from other countries. This may have resulted in over- or underestimation of the incidence for some countries. We addressed this uncertainty by estimating 95% CIs (for the community incidence in the <5 and 5–9 year olds), or by providing a minimum-maximum range otherwise. We did not consider immigration in our estimates. A seroprevalence study carried out in adults in the Netherlands [64] showed that immigrants, in particular first generation immigrants, were more likely to be varicella seronegative compared to Dutch-born adults. Studies in the UK, Ireland and Spain among pregnant women have also shown that foreign-born women are more likely to be susceptible to varicella [36, 44, 47, 57]. There is a lack of studies addressing the impact of immigration on varicella epidemiology. Most seroprevalence surveys are carried out using residual serum samples with no information on immigration status [41]. As evidenced by van Rijckevorsel et al. [64], varicella serological profiles may show geographical differences within countries, with urban areas (being areas where immigration is typically concentrated) often presenting a higher proportion of varicella susceptible adults. To estimate varicella mortality, we used data from the WHO DMDB [11]. Mortality causes are coded using International Classification of Diseases (ICD)-9 or ICD-10 and it is difficult to ascertain the accuracy of the coding. Hence misclassification of the cause of death cannot be excluded. This may have resulted in over or underestimation of varicella mortality.

Given the limited number of studies that looked at specific complications and/or sequelae, we did not model these outcomes separately. It is however noteworthy that in addition to the immediate burden on the health care system as discussed in this paper, varicella may also cause complications and long term sequelae. In children (0–17 years), reported incidence of varicella complications requiring hospitalisation ranges from 0.82 per 100,000 of the population in the UK and Ireland [119], to 19 per 100,000 in Belgium [75]. Differences in incidence are probably related to differences in the definitions used for complicated varicella. In the UK study [119] only severe complications were included, excluding secondary skin infections, while the Belgian study [75] included any complication. The most frequent complications reported were bacterial superinfections, followed by varicella pneumonia and neurological complications. Concerning long term sequelae, these are usually a result of neurological complications of varicella. Long term sequelae have been reported in 0.4 to 8% of children hospitalised for varicella [85, 95, 100, 103, 130].

Recent published data from Norway provided us with an opportunity to validate the methodology used. We found that our model overestimated the varicella community incidence in Norway in <5 year olds and underestimated the incidence in 5 to 9 year olds. There were small differences in the predicted vs. observed incidence for ages 10 to 39, with the model underestimating the incidence in the ≥40 year olds. The model we used to estimate community incidence was based on the prediction variables including the percentage of children <3 years of age receiving formal childcare and population density. While for both prediction variables Norway is similar to northern European countries with the highest incidence observed in children <5 years old, the varicella epidemiology pattern in Norway also somewhat resembles that of southern European countries with a relatively high observed incidence in the 5 to 9 year olds. Although we explored the inclusion of additional explanatory variables in our model, none of them improved the fit of the model. The methodology we used to estimate PCI overestimated the observed PCI in Norway, particularly in the younger age groups. We used the minimum-maximum observed PCR to estimate PCI in countries without data. Health care seeking behaviour, and hence the PCR, varies strongly between countries [104]. For example, 38% of children <5 years with varicella consulting a physician [131] in the Netherlands compared to 88% of children <3 years in France [132]. There may also be differences in hospitalisation policies for varicella cases between countries which potentially affect the hospitalisation rate. We tried to address this uncertainty by estimating a minimum-maximum range for PCI and HI. However, we cannot exclude that PCI and HI may have been over or underestimated for some countries, like in the case of Norway.

## Conclusions

In conclusion, the estimated burden of varicella in Europe in the pre-immunization period was substantial with more than 5 million new cases estimated annually, of which slightly more than half is expected to lead to a physician consultation, about 20,000 to hospitalisation, and up to 80 to death. Since very few countries or regions have introduced universal childhood varicella immunization programs, these figures are probably still true today. Although the main share of the burden is in children <5 years old, adults require hospitalisation more often and are at higher risk of death. This information should be considered when planning and evaluating varicella control strategies. Improving and standardizing varicella surveillance in Europe, as initiated by ECDC, will be important to improve the quality of data available and allow better inter-country comparison. There is also a need to better understand the driving factors of country-specific differences in varicella transmission and health care utilization. In addition, future research on sero-epidemiology with prospective sampling and data collection, ensuring the inclusion of migrant populations, would further improve our understanding of the epidemiology of varicella in Europe.

## Additional files

Additional file 1: Risk of bias assessment tool. (DOCX 15 kb)

Additional file 2: Variables used in the linear regression models. (DOCX 13 kb)

Additional file 3: Study characteristics. (XLSX 12 kb)

Additional file 4: Data extraction tables. (XLSX 308 kb)

Additional file 5: Country and age-specific number of varicella cases in the community, primary care visits, hospitalizations and deaths. (XLSX 24 kb)

## Abbreviations

CI: Confidence interval
DMDB: Detailed Mortality Database
ECDC: European Centre for Disease Prevention and Control
EMA: European Medicines Agency
EU: European Union
HI: Hospitalisation incidence
HR: Hospitalisation rate
PCI: Primary care incidence
PCR: Primary care rate
SDC: Supplementary Digital Content
SLR: Systematic literature review
WHO: World Health Organisation
