# Supplementary material for: Estimation of the burden of varicella in Europe before the introduction of universal childhood immunization
Source: BMC Infect Dis. 2017 May 18;17:353. doi: 10.1186/s12879-017-2445-2 (PMC5437534; doi:10.1186/s12879-017-2445-2)
Supplement: Supplementary file 1 — Risk of bias assessment tool. (DOCX 15 kb) [file 12879_2017_2445_MOESM1_ESM.docx]

Supplement 1. Risk of bias assessment tool

Modified from Hoy et al. Assessing risk of bias in prevalence studies: modification of an existing tool and evidence of interrater agreement. J Clin Epidemiol 2012; 65: 934 – 939.

| Risk of bias item | Criteria for answers |
| --- | --- |
| 1. Was the study’s target population a close representation of the national population in relation to relevant variables, e.g. age, sex, occupation? | - Yes (LOW RISK) - No (HIGH RISK) |
| 2. Was the sampling frame a true or close representation of the target population? | - Yes (LOW RISK) - No (HIGH RISK) |
| 3. Was some form of random selection used to select the sample, OR, was a census undertaken? | - Yes (LOW RISK): a census was undertaken OR some form of random selection was used (eg. Simple random sampling, systematic sampling…) - No (HIGH RISK) |
| 4. Was the likelihood of non-response/non-participation bias minimal? | - Yes (LOW RISK): response rate was 75% or higher OR an analysis was performed that showed no significant difference in relevant demographic characteristics between responders and non-responders - No (HIGH RISK) |
| 5. Was an acceptable case definition used and applied to all subjects? | - Yes (LOW RISK) - No (HIGH RISK) |
| 6. Was the same mode of data collection used for all subjects? | - Yes (LOW RISK) - No (HIGH RISK) |
| 7. Was the length of the shortest prevalence/incidence period for the parameter of interest appropriate? | - Yes (LOW RISK): The shortest prevalence period for the parameter of interest was appropriate (e.g. point prevalence, one-week prevalence, one-year prevalence). - No (HIGH RISK): The shortest prevalence period for the parameter of interest was not appropriate (e.g. lifetime prevalence) |
| 8. Were the numerator(s) and the denominator(s) for the parameter of interest appropriate? | - Yes (LOW RISK) - No (HIGH RISK) |
| 9. Total score | - Number of YES responses in items 1 - 8 |
| 11. Summary item on the overall risk of study bias | - LOW RISK OF BIAS: Further research is very unlikely to change our confidence in the estimate - MODERATE RISK OF BIAS: Further research is likely to have an important impact on our confidence in the estimate and may change the estimate - HIGH RISK OF BIAS: Further research is very likely to have an important impact on our confidence in the estimate and is likely to change the estimate |
