# Supplementary material for: Estimation of the burden of varicella in Europe before the introduction of universal childhood immunization
Source: BMC Infect Dis. 2017 May 18;17:353. doi: 10.1186/s12879-017-2445-2 (PMC5437534; doi:10.1186/s12879-017-2445-2)
Supplement: Supplementary file 2 — Variables used in the linear regression models. (DOCX 13 kb) [file 12879_2017_2445_MOESM2_ESM.docx]

| **Indicator** | **Data source** | **Year** | **Comments** |
| --- | --- | --- | --- |
| Inequality of income distribution (ratio 20% richest/20% poorest) | Eurostat | 2014 |  |
| People at risk of poverty after social transfers (%) | Eurostat | 2014 |  |
| % children < 3 y that receive no formal childcare | Eurostat | 2014 |  |
| Total health expenditure (as % of GDP) | World Bank | 2014 |  |
| % of households with 1, 2, 3 and 4 or more children | Eurostat | 2014 |  |
| Number of annual consultations of a medical doctor per inhabitant | Eurostat | 2013 | Greece, Spain (2006)  UK (2009)  Ireland (2010)  Belgium (2011)  Portugal, Switzerland (2012) |
| Number of annual acute hospital discharges per 100 population | Eurostat | 2013 | Denmark (2009)  Greece, Netherlands (2010)  France (2011)  Hungary, Belgium, Sweden, Malta, Latvia (2012) |
| Population density (per square kilometer) | Eurostat | 2014 |  |

Supplement 2. Variables used in the linear regression models
